# MR guided right heart catheterization - the NIH experience

**DOI:** 10.1186/1532-429X-17-S1-O20

**Published:** 2015-02-03

**Authors:** Toby Rogers, Kanishka Ratnayaka, Annette Stine, Laurie Grant, William Schenke, Jonathan R Mazal, Michael Hansen, Anthony Z Faranesh, Robert J Lederman

**Affiliations:** 1 National Heart Lung and Blood Institute, National Institues of Health, Bethesda, MD, USA; 2 Department of Cardiology, Children's National Medical Center, Washington, DC, USA

## Background

Realtime MR enables radiation free guidance for right heart catheterization (RHC). In addition to catheter navigation for sampling of invasive pressures and blood oxygen saturations, MR permits concomitant assessment of cardiac chamber volumes and cardiac output with phase contrast flow measurements. By performing repeat measurements under different physiological provocations (e.g. saline volume challenge, inhaled nitric oxide, or exercise), diagnostic yield increases by revealing symptoms and pathologic findings not apparent at rest. Herein we present the NIH experience of MR RHC to date.

## Methods

**Figure 1:**
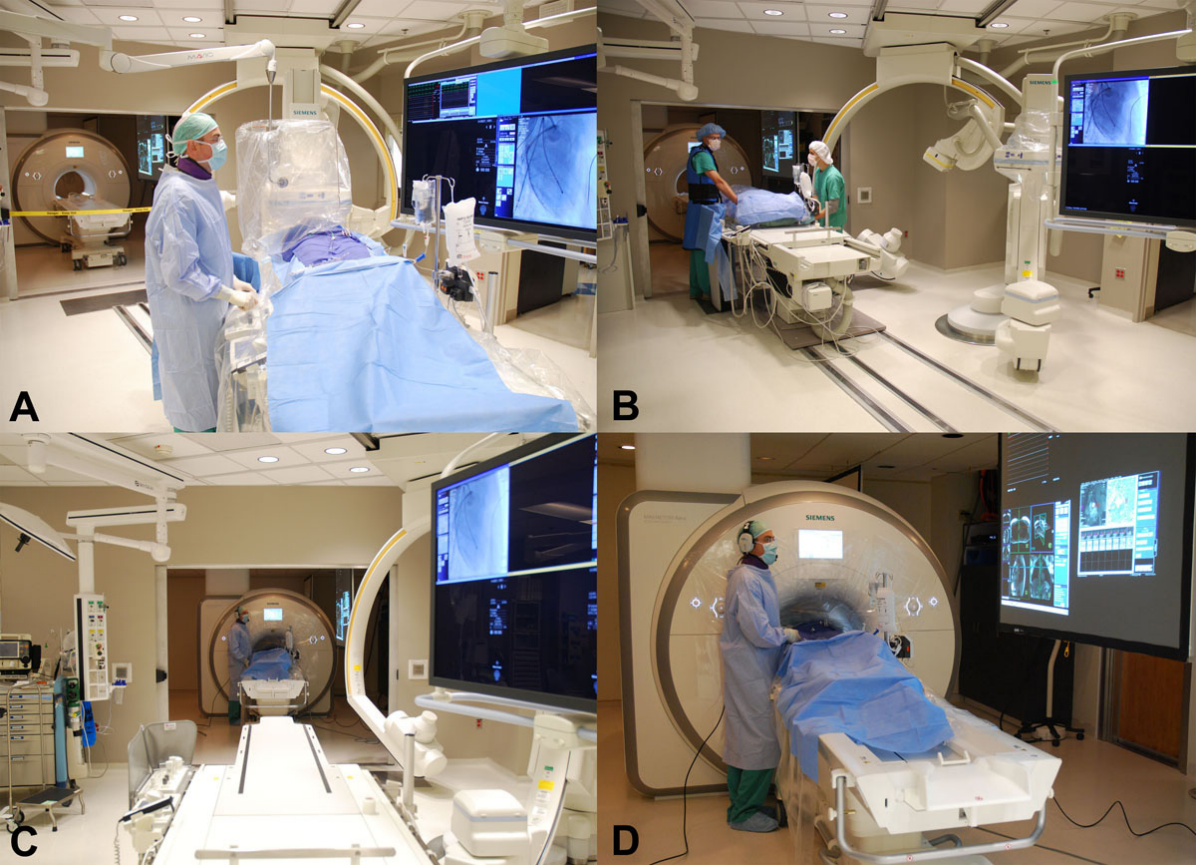
(A) If required X-ray coronary angiography is performed before MR RHC. (B) The patient is transferred to MRI maintaining sterility of the field. (C) View from X-ray into the MR room. (D) MR RHC performed from the femoral vein. Images are projected onto a screen for the operator. Noise-suppression headsets are used for communication between staff and with the patient.

**Figure 2:**
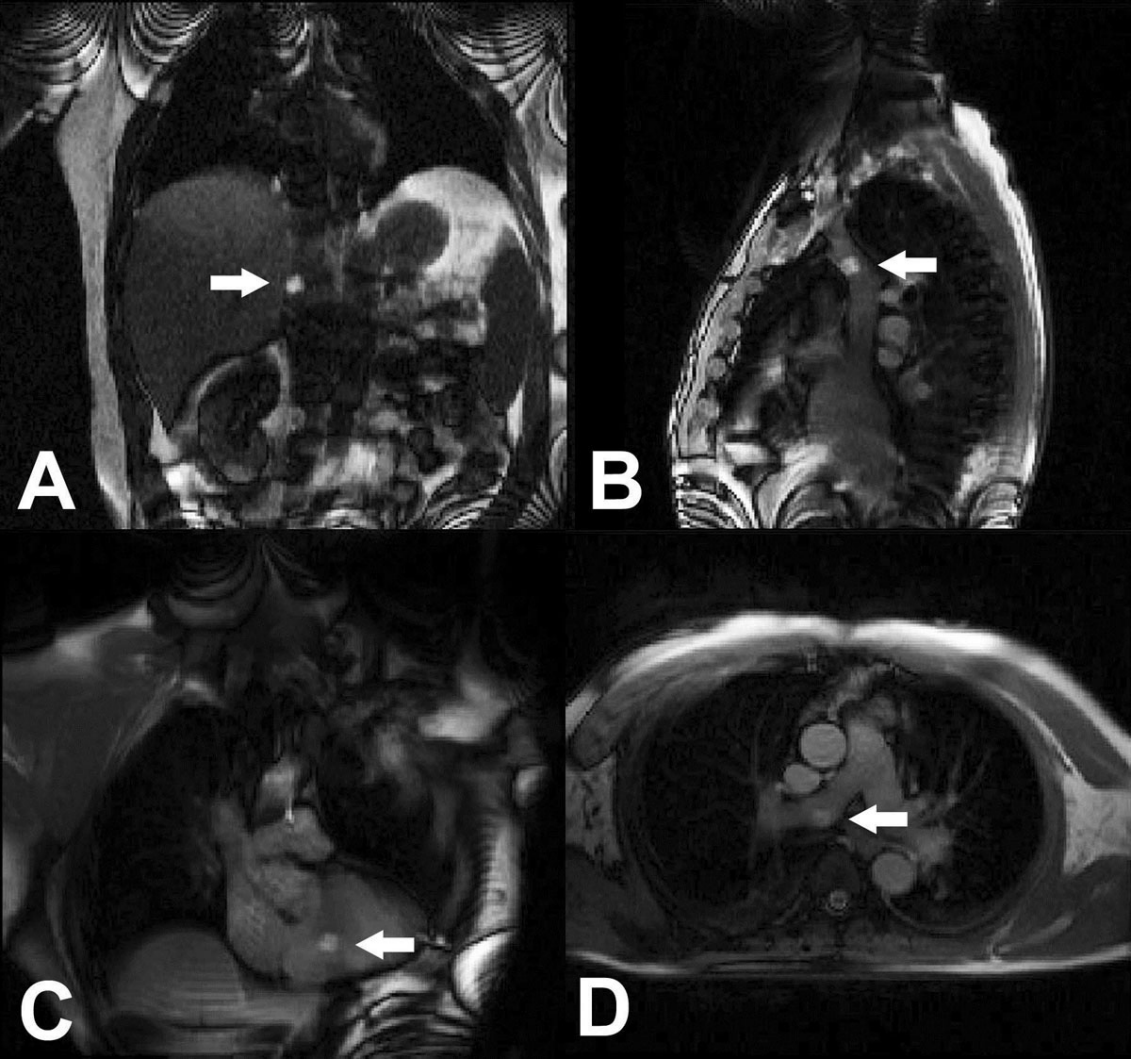
Gadolinium-filled balloon at the tip of the catheter (arrow) in the (A) inferior vena cava, (B) superior vena cava, (C) right ventricle, (D) right pulmonary artery.

All consecutive patients referred for invasive left or right heart catheterization at our institution were invited to undergo MR RHC unless a clear contraindication was present. Informed consent was obtained. Vascular access was obtained outside the MR room and patients were transferred into the MR bore (Figure 1). After baseline MR scanning, a gadolinium-filled balloon-tip end hole catheter was navigated through the right heart under realtime MR guidance (Figure 2). Measurements were repeated under physiological provocation with saline challenge or inhaled nitric oxide in latter patients.

## Results

79 patients consented and, after exclusions, 72 patients underwent MR RHC. Median age was 56yrs (range 26-83yrs) and 51% were female. RHC was completed with MR guidance only in 96% of patients. Only 3/72 required a guidewire and X-ray guidance to complete the procedure, all of which occurred early in our experience. Median procedure time from sheath entry to exit was 26min (range 11-63min). There was a definite learning curve, which permitted completion of repeated measurements under additional physiological provocation with a small increase on total procedure time (mean procedure time 23min vs. 38min, for the first 36 vs. the last 36 patients respectively). No serious complications occurred in any patient.

## Conclusions

We have demonstrated that a comprehensive RHC can be performed under MR guidance in almost all patients without the need for additional X-ray imaging. Furthermore, with procedural streamlining, it is possible to perform a comprehensive baseline examination followed by repeat measurements under physiological provocation in under 40min. Superior clinical information is obtained and as a result, MR RHC has been reclassified as a standard clinical procedure at our institution.

## Funding

This work was supported by the Division of Intramural Research, National Heart Lung and Blood Institute, National Institutes of Health (Z01-HL005062).

